# The Letter of Medico-Chirurgus on "A New Set of Physicians"

**Published:** 1826-07

**Authors:** 


### MEDICAL JURISPRUDENCE.*

The complaint of Medico-Chirurgus, though very evidently drawn from a single and local instance, would apply to every town, from the metropolis downwards—and not to the stigmatised Scotch Diplomatists only, but to men *melioris notæ*—not of the present time alone, but for more than a century back. It is true the profession is over-stocked—and men, the young as well as the old, will descend to many a shift, rather than starve. The poor apothecary consented to give Romeo the poison, from necessity rather than inclination:—the poor half-pay surgeon,

* A letter addressed to the Medical Profession, on the Encroachments on the Practice of the Surgeon-Apothecary, by a New Set of Physicians. By Medico-Chirurgus. 8vo. sewed, p. 20.

who served his country during a long and disastrous war, and wasted his prime of life, "by flood and field," cannot live, still less support wife and children on six shillings a day. He must endeavour to turn his experience to some account, and pick up a fee in town or village, as he can. Medico-Chirurgus is too severe upon this class. It is too late in life for many of them to begin as general practitioners,—and still more so to go to school again, and relearn all the trash, and nonsense, and theory, and errors, which every man is glad to forget in his journey through life, but which alone would enable him to maintain a *thesis* at the age of 40 or 50, and thus come out—"privilegiis *ritè* ac *legitimè* consequendis." If Medico-Chirurgus himself, after having got a good education, and practising for 30 years, chose to take out an Aberdeen Diploma, and restrict himself to the practice of physic in his native or a neighbouring town, would any man of sense or liberality say that he was unqualified for such a situation? These are the two classes of men who take such degrees; and the state of society, and the circumstances of the case, justify the deviation from the regular path. With any other description of medical men the said degree would be of no use, and certainly should not be tolerated. So far then, we think the complaint of Medico-Chirurgus is not founded on justice and liberality:—not so his animadversions on the *conduct* of individuals who disgrace the profession by intriguing and manœuvring to work themselves into the favour of families, at the expense of the general practitioner previously, or at the time, in attendance. The author of the pamphlet before us cannot hold in greater detestation than we do, such a vile mode of rising in the world. But, alas! Medico-Chirurgus knows little of the world, if he thinks this stigma is confined entirely to diplomas signed north of the Frith of Forth!—Many "*ritè et legitimè*" doctors, on both sides of the Tweed, have taken out the degree of "Master of Arts," in the valuable science of *supplantation*. They do not, indeed, go to work in the clumsy country-bumpkin way which our author describes.

"Presuming on having practised the different branches of the profession, a physician of this cast is at one time found dressing a sore leg, or attempting to reduce a fracture, and at another officiously interfering in a lying-in chamber; which perhaps he enters for the first time in his life. No set of men are more clamorous for practice, and none less delicate in their mode of obtaining it; and that they succeed beyond their merits, is a truth that must be acknowledged. They wish to inculcate an opinion that they have received from nature, a secret propensity to all that is good and virtuous; and indulge the extravagant vanity, that they are, by nature, superior to all others. They have the effrontery to expect that, when they are consulted, the inferior practitioner, as they indecently style the family attendant, is to surrender the case entirely into their hands; and express great surprise when they find any man with sufficient good sense and regard for the welfare of his patient, not tamely to submit to their wishes. Inflated with a vain conceit of their acquirements, with an intolerant temper, they aim at universal dominion over their better-informed and more deserving brethren; and varnish over their mean designs with an affected liberality." 9.

No, no! This would only do among mountains or fens. The master of arts has a far more refined mode of procedure. By means of the fifth pair of nerves, and the portio dura of the auditory, the knowing physician—aye, and the pure surgeon too, will "damn with faint praise," or without speaking a single word, the general practitioner before his face!—This, indeed, is the acmé of perfection in the science of supplantation. But as, "in our father's house there are many mansions," so, in this noble art there are various degrees of attainment. There are many who cannot perform in *duetts*, but who are excellent at *solos* with the patient or friends. Some, indeed, have the organ of supplantation so prominently developed, that the general practitioners are all warned of their danger, and a perpetual warfare is waged by the two parties—one endeavouring to *keep*, and the other to *get* his enemy out! This, of course, brings great odium and discredit on the profession—and causes great misery and dissatisfaction among patients and their friends. The following graphic sketch is, we know, from Nature.

"His next care is to become acquainted with the general practitioner in the most extensive practice, and by one art or another he prevails on him to introduce him first to his friends, and by degrees to his patients. At first he is all attention and kindness, but as his introductions increase, and he finds himself on firmer ground, his next care is to detach the patients of his unsuspecting friend, and lessen that confidence which he has long and deservedly enjoyed. This may not appear an easy task, but perseverance overcomes many difficulties; and when a man of this character once obtains a footing in a family, he will never rest until he has succeeded in supplanting their regular attendant. One mode of advantage is, that, having much leisure time, whilst the general practitioner is engaged in earning his daily bread, he will take frequent opportunities of making his professional visits in the absence of the surgeon, and by insinuation and other arts lessen his value and importance. The family will compare the extreme attention of the physician, with the occasional visits of the surgeon, who, for the first time, hears something like disapprobation, and hints of neglect—these gradually increase, and, in the end, the family is too often lost to the old attendant, and taken possession of by this notable physician, who reconciles them with the assurance, that his prescriptions can be very accurately dispensed by a neighbouring druggist, with whom he probably shares a profit on the medicine, a practice as notorious as the sun at noon-day." 7.

We hope and trust that the last part of the charge—the practice of participation with the druggist, is of very limited extent; but that the system of detraction prevails to a considerable degree, especially among the lower and more necessitous orders of the profession—and, we are sorry to say, among a *few*, whose rank, riches, and established reputation leave them no excuse for such conduct, we are compelled to confess. Nothing has tended more to injure the profession in the eyes of the public than this intestine illiberality among its own professors—and we greatly fear that there will always be found individuals who, caring only for their own private interests, and totally indifferent to the honour and respectability of the faculty to which they belong, will give way to the baser passions of human nature, and slander others with the view of benefitting themselves. It is the duty, therefore, of the influential men of the profession to discountenance such men and such conduct by every mark of detestation which it is in their power to express. Heaven knows there are sufficient sources of error, uncertainty, and difficulty, in the practice of medicine, without aggravating them by malicious insinuations and ungenerous comments, that too often destroy the peace of mind of suffering patients, surviving relatives, and the reputation of medical men, without a shadow of justice or of benefit to any party ! Patients and friends are but too apt to indulge in reflexions on the skill or success of their medical attendants, and it is the duty of all professional men immediately to check such querulous insinuations, instead of listening to them, or fostering the spirit of discontent thus evinced. If medical men were to protect each other's reputation when clandestinely attacked, they would all reap the benefit, and they would rise higher in public estimation.

But it will be said—indeed, we have heard it said—" are we to conceal the ignorance, screen the blunders, and sanction the errors of our brethren ?" Who has constituted us their judges, we ask ? Are we to listen to the *exparte* statements of patients or friends, whose passions are enlisted in the cause of their fancied wrongs—and thus pass judgment on hearing one side of the question ?—Such decisions would be disgraceful to a Turk—or even to a savage—and yet such decisions are daily given by one medical man against another ! Vice, they say, is a monster of such horrible aspect, that it needs but to be seen, to be dreaded. No vice can be more degrading, disgusting, and injurious to medical society than the spirit of detraction which we have thus portrayed. We conjure our brethren to shake off a stigma from the medical character, which is become proverbial by its universality.

